# Association of cognitive function with glucose tolerance and trajectories of glucose tolerance over 12 years in the AusDiab study

**DOI:** 10.1186/s13195-015-0131-4

**Published:** 2015-07-12

**Authors:** Kaarin J. Anstey, Kerry Sargent-Cox, Ranmalee Eramudugolla, Dianna J. Magliano, Jonathan E. Shaw

**Affiliations:** Centre for Research on Ageing, Health and Wellbeing, Australian National University, 54 Mills Road, Canberra, ACT 0200 Australia; Baker IDI Heart and Diabetes Institute, PO Box 6492, Melbourne, Victoria 3004 Australia

## Abstract

**Introduction:**

We investigated the association between glucose tolerance status and trajectories of change in blood glucose, and cognitive function in adults aged 25 to 85.

**Methods:**

The sample (n = 4547) was drawn from a national, population-based cohort study in Australia (AusDiab). Fasting plasma glucose (FPG), glycated haemoglobin (HbA1c) and general health were assessed at 0, 5 and 12 years. Covariates included age, education, body mass index, blood pressure and physical activity. At 12 years, participants completed assessments of memory, processing speed and verbal ability.

**Results:**

Known diabetes at baseline was associated with slower processing speed at 12 years in both younger (25–59 years) and older (>60 years) age-groups. After 12 years of follow-up, adults aged < 60 with diabetes at baseline had a mean speed score of 49.17 (SE = 1.09) compared with 52.39 (SE = 0.20) in normals. Among younger males without diagnosed diabetes, reduced memory at 12 years was associated with higher HbA1c at 5 years (β = −0.91, SE = 0.26, p < 0.001). No effects were apparent for females or older males. Adjusting for insulin sensitivity (HOMA-%S) and hs-C reactive protein attenuated these associations, but depression and CVD risk did not. Latent class analysis was used to analyse the associations between trajectories of HbA1C and glucose over 12 years, and cognition. Identified classes were described as 1) normal and stable blood glucose over time (reference), 2) high intercept but stable blood glucose over time, and 3) increasing blood glucose over time. In both young males and females, high stable glucose measures were associated with poorer cognitive function after 12 years.

**Conclusion:**

Those with type 2 diabetes, younger males with high non-diabetic HbA1c, and adults with high stable blood glucose are at increased risk of poorer cognition. The findings reinforce the need for management of diabetes risk factors in midlife.

**Electronic supplementary material:**

The online version of this article (doi:10.1186/s13195-015-0131-4) contains supplementary material, which is available to authorized users.

## Introduction

Recent research on both middle-aged adults [1] and older adults [2] has shown that type 2 diabetes increases the risk of late-life cognitive decline, Alzheimer’s disease (AD) and all-cause dementia [3–5]. Some authors have argued that the critical period for the effect of type 2 diabetes on cognition is in late life [6], rather than middle age. Recent data, however, show associations between type 2 diabetes and mild cognitive impairment (MCI) in middle age (50–65 years old) [7], and between type 2 diabetes and cognitive decline in mid-life [8]. There is also growing evidence that high normal blood glucose is associated with cognitive decline. Studies report a direct association between glycaemia and risk of dementia in older adults with and without diabetes [3], as well as associations between poor glucose metabolism and reduced executive function in mid-life [9], between insulin resistance and brain atrophy in middle age [10], and between high fasting blood glucose and lower memory scores, hippocampal atrophy and reduced hippocampal microstructure in older adults (≥60 years old) without diabetes [11, 12].

Current understanding of the role of glycaemic control and ageing in the transition to cognitive impairment is limited by a lack of longitudinal data on the relationship between blood glucose and cognition. One recent study found that longitudinal trajectories of glycated haemoglobin (HbA1c) in older adults with type 2 diabetes (*N* = 835) predict cognitive performance, specifically executive function [13]. The only study to our knowledge that has examined longitudinal change in HbA1c and cognition in those with normal glucose tolerance (NGT) [14] did so in a sample of older adults (≥75 years old, *N* = 101) and found a decrease of 1.37 Mini-Mental State Examination (MMSE) points per 1 % increase in HbA1c. Analysis of the Whitehall II mid-life cohort found that cognitive change in prediabetes and new diabetes was not different from that in NGT [15].

Data on the relationship between glucose tolerance and cognition in mid-life, particularly in prediabetic stages, are sparse [5]. We therefore evaluated how glucose tolerance status, fasting plasma glucose (FPG) and HbA1c over 12 years were associated with cognitive abilities including processing speed, memory and verbal ability in adults aged 25–59 and 60–85. We hypothesised that type 2 diabetes and glycaemia would be associated with poorer cognition in both younger and older age groups and with increased risk of cognitive impairment in the older group. Our cohort was assessed at 0, 5 and 12 years on measures of glycaemia and diabetes status, and cognition was assessed at 12 years.

## Methods

### Study population

The Australian Diabetes, Obesity and Lifestyle Study (AusDiab) is a national population-based survey undertaken to determine the prevalence and incidence of diabetes, obesity and other cardiovascular disease risk factors in Australian adults. The baseline study was conducted in 1999–2000 and involved 11,247 adults aged 25 years and above, from across Australia. Sample selection involved drawing a stratified cluster sample from 42 randomly selected census collector districts across Australia. Details of the sampling process have been described elsewhere [16]. Information was collected using a brief household interview, followed by a biomedical examination. Among those who completed the household interview, the response rate for the baseline biomedical examination was 55 % (*n* = 11,247). The first follow-up (*n* = 6537) was conducted in 2004–2005 (i.e., 5-year follow-up), where all living eligible participants were invited to attend the follow-up. Participants from the baseline survey were classed as ineligible for follow-up if they refused contact, were deceased, had moved overseas, were in high-level nursing care or reported a terminal illness. The second follow-up was conducted in 2011–2012. Of the 4614 participants who attended the interview, cognitive assessment was conducted on 4562 adults. At each follow-up, information was collected on demographics, medical and family history, lifestyle risks including level of physical activity, diet, alcohol and tobacco consumption, physical measurements, and blood and urine measurements. Cognitive assessment was conducted at the second follow-up only (i.e., 12-year follow-up).

### Standard protocol approvals and participant consent

Written informed consent for research was obtained from all participants in the study, and the research protocol was approved by the human research ethics review boards of the International Diabetes Institute, Monash University and the Alfred Hospital, Melbourne.

### Diabetes status and blood glucose measures

All participants except for those currently receiving treatment for diabetes and those who were pregnant underwent a standard 75 g oral glucose tolerance test (OGTT) [17]. At baseline, the FPG and 2-hour plasma glucose (PG) levels were determined by a glucose oxidase method using an Olympus AU600 automated analyser (Olympus Optical, Tokyo, Japan). At Wave 2, a spectrophotometric hexokinase method using a Roche Modular (Roche Diagnostics, Indianapolis, IN, USA) was used. At Wave 3, a hexokinase method using a Siemens Advia 2400 (Siemens AG, Munich, Germany) was used. Procedures for comparing results from the different assays are described elsewhere [18]. Total HbA1c analysis used high-performance liquid chromatography (Bio-Rad Variant Hemoglobin Testing System; Bio-Rad, Hercules, CA, USA) with standardised conversion to A1c values. High-sensitivity C-reactive protein (hs-CRP) was measured in stored samples (−80 °C) via a two-site chemiluminescent immunoassay (BioMediq IMMULITE 2000; CA, USA).

Glucose tolerance status was classified according to the 1999 World Health Organization criteria [17]. Briefly, participants were classified as having ‘known diabetes’ (KDM) if they reported having doctor-diagnosed diabetes and were either taking hypoglycaemic medication or had FPG ≥7 mmol/l or 2-hour PG ≥11.1 mmol/l. Participants not reporting diabetes but with FPG ≥7 mmol/l or 2-hour PG ≥11.1 mmol/l were classified as having ‘newly diagnosed diabetes’ (NDM). For participants not reporting diabetes, impaired glucose tolerance (IGT) was classified with FPG <126 mg/dl and 2-hour PG between 140 and 199 mg/dl. Impaired fasting glucose (IFG) was classified if FPG was between 110 and 125 mg/dl and 2-hour PG <140 mg/dl. NGT was indicated by both FPG <110 mg/dl and 2-hour PG <140 mg/dl. Insulin sensitivity was estimated from FPG and fasting insulin concentrations using Homeostatic model assessment (HOMA-2), which was calculated with the HOMA-2 program [19].

### Anthropometry and blood pressure

Height and weight measures were taken at each wave, from which the body mass index (BMI) was calculated. Blood pressure measurements at Waves 2 and 3 were performed (while seated after 5 minutes rest) using an automated monitor (Dinamap Pro-Series Monitor Model DP 101-NIBP, pulse and recorder; GE Medical Systems, Freiburg, Germany). The mean of the first two measurements were taken. At baseline, blood pressure was measured in the same manner for all states except Victoria, where a manual sphygmamometer was used. These measures were then adjusted to be comparable with automated methods. Mean arterial blood pressure (MAP) was calculated as [20]:

$$ \mathrm{MAP} = \mathrm{diastolic} + \left(1/3 \times \left(\mathrm{systolic}\ \hbox{--}\ \mathrm{diastolic}\right)\right) $$

### Physical activity

The level of physical activity was determined using questions relating to time (in minutes) spent walking and time spent doing vigorous and moderate physical activity in the previous week [21].

### Cognitive measures

Memory was assessed with the California Verbal Learning Test (CVLT) [22]. In this test, participants listened to a list of 16 common shopping list items read by the interviewer, and subsequently repeated as many of these items as possible (immediate recall). Repeat learning trials were not administered. After a delay of 20 minutes filled with other non-verbal survey items, the participant was asked to recall the list a second time (delayed recall). Scores range from 0 to 16. Vocabulary and verbal knowledge were assessed using a lexical decision task, the Spot-the-Word (STW) test [23]. In this test, participants were presented with 60 pairs of words. One was a real word and one a non-word, and the participant was required to identify the real word. Scores range from 0 to 60. Performance on this task correlates with verbal knowledge and is relatively resistant to age-related decline. Processing speed was assessed with the Symbol–Digit Modalities test (SDMT) (oral version) [24]. Participants used a reference key to find and report the numbers corresponding to a series of geometric figures as quickly as possible. The score ranged from 0 to 60 and represents the number of correct responses given within a 90-second period. A global screening test for cognitive impairment, the MMSE [25], was also included and administered to individuals aged above 40 years, with scores below 24/30 indicating cognitive impairment.

### Statistical analyses

Comparisons between those lost to follow-up or who did not complete the cognitive tests and those included in the study were conducted using *t* tests and chi-square tests. Individuals reporting type 1 diabetes were excluded from analyses leading to a final sample of 4547, all with a follow-up time of 12 years. Generalised linear models (GLM) were used to estimate the associations of diabetes status at baseline and 5 years, with cognitive function at 12 years, using case-wise exclusion for missing data. In the second set of analyses, participants with KDM at any wave or NDM at the 12-year follow-up were excluded, leaving a sample of 3515 participants. GLM were used to estimate the association of glucose and HbA1c at baseline, 5 years and 12 years with cognitive function at 12 years, with case-wise exclusion for missing data. Covariates included age, education, BMI, smoking, MAP and exercise. Analyses were stratified by sex and age group (25–59 years, 60+ years). A significance level of *p* <0.01 was used for interpretation of results. In the final set of analyses, latent class analysis was used to identify patterns of change in FBG and HbA1c over 12 years. Classes of change (stable normal, stable high normal, increasing) were then examined in relation to cognitive outcomes at 12 years using GLM.

## Results

The analytic sample comprised 4547 participants (2032 men) with cognitive data at the third wave of follow-up. Compared with the AusDiab sample at recruitment (*N* = 11,247), the study sample did not differ in gender distribution but was younger (mean 48.9 vs. 53.3 years, *p* <0.001), had lower BMI (26.6 vs. 27.3, *p* <0.001), spent more time exercising (285 vs. 266 minutes, *p* <0.01), was less likely to smoke (*p* <0.01) had higher levels of education (*p* <0.001) and had lower FPG (5.5 vs. 5.7, *p* <0.001) and HbA1c (5.2 vs. 5.3, *p* <0.001). However, loss to follow-up and attrition due to death meant that the analytic sample was healthier than the baseline sample. Sample attrition was 37 % at 5 years and 35 % at 12 years. Attrition due to death was 9.5 % and 15 % at each follow-up respectively. Analysis of glucose tolerance status as a function of death status at 12 years (see Additional file 1) indicated that those who had died prior to the third wave of data collection were more likely to be classified as KDM and IGT, and less likely to be classified as NGT at baseline (*χ*^*2*^ (4) = 510.94, *p* <0.001) and at 5 years (*χ*^2^ (4) = 97.35, *p* <0.001). They also had higher mean HbA1c at baseline (*F*(1, 11143) = 330.71, *p* <0.001) and at 5 years (*F*(1, 6438) = 60.80, *p* <0.001), and higher mean FBG at baseline *F*(1, 11209) = 295.66, *p* <0.001) and at 5 years (*F*(1, 11209) = 295.66, *p* <0.001). Of the sample used in this study, 95 participants had KDM at baseline, 169 at the 5-year follow-up and 310 at the 12 year follow-up. There were 77 cases of incident diabetes at the 5-year follow-up (one KDM case reverted to IGT) and 111 incident cases at the 12-year follow-up (nine cases reverting to IGT, IFG, new diabetes or NGT).

### Participant characteristics

The participant characteristics are shown in Table 1 by glucose tolerance status at baseline. The NGT group was younger and scored higher on all cognitive measures except STW. NDM differed from the other diabetes status groups with regard to exercise. Participant characteristics also differed between the sexes (Additional file 1). Within the NGT group, males (relative to females) had higher systolic blood pressure, higher diastolic blood pressure, higher BMI, higher FPG, higher HbA1c and were more likely to have smoked, but did more exercise. There were no sex differences in terms of age, verbal ability or education.

**Table 1:** Baseline characteristics and cognition at 12 years according to glucose tolerance status at baseline

	NGT	IFG	IGT	NDM	KDM
	(*N* = 3606)	(*N* = 254)	(*N* = 428)	(*N* = 119)	(*N* = 94)
Age range	25–83	25–83	25–85	25–79	36–75
Age, mean (SD)	47.71 (11.04)	51.31 (10.6)*	53.99 (11.1)*	56.6 (10.5)*	56.4 (9.4)*
Female (%)	2060 (57.1)	65 (25.6)*	249 (58.2)	56 (47.1)	48 (44.0)
BMI, mean (SD) (kg/m^2^)	25.9 (4.3)	28.5 (4.1)*	28.9 (5.3)*	30.1 (5.5)*	30.6 (6.0)*
Waist circumference, mean (SD) (cm)					
Female	81.8 (11.6)	91.7 (12.4)*	91.0 (13.6)*	96.05 (14.2)*	98.7 (14.0)*
Male	95.1 (10.2)	100.8 (10.4)*	100.2 (10.8)*	103.7 (11.7)*	104.7 (14.2)*
Highest level of education (%)					
Secondary school	1138 (31.7)	86 (34.3)	174 (40.7)*	48 (41.0)	39 (36.0)
Trade certificate	1055 (29.4)	91 (36.3)	115 (26.9)	38 (32.5)	37 (34.3)
Tertiary	1397 (38.9)	74 (29.5)*	138 (32.3)	31 (26.5)	32 (29.6)
Current smoker (%)	390 (11.0)	38 (15.1)	33 (7.8)	7 (6.0)	11 (10.1)
Ex-smoker (%)	960 (27.0)	92 (36.7)*	149 (35.1)*	39 (33.3)	42 (38.5)
Blood pressure, mean (SD) (mmHg)					
Systolic	124.42 (15.7)	130.97 (16.3*	134.06 (17.2)*	143.19 (19.7)*	141.58 (19.9)*
Diastolic	69.10 (11.1)	73.80 (11.2)*	72.31 (11.7)*	77.40 (11.7)*	74.80 (11.1)*
Fasting blood glucose, mean (SD) (mg/dl)	94.41 (7.0)	113.69 (3.6)	100.54 (10.1)*	131.17 (31.2)*	171.71 (66.7)*
HbA1c, mean (SD) (%)	5.04 (0.2)	5.29 (0.26)*	5.27 (0.3)*	5.90 (1.0)*	7.30 (1.6)*
Exercise mean (SD) (minutes/week)	295.0 (338.9)	275.5 (335.4)	216.02 (260.7)*	236.14 (290.0)*	301.9 (357.6)
Cognition at 12 years, mean (SD)					
MMSE score	28.14 (1.94)	27.6 (2.19)*	27.6 (2.4)*	26.9 (3.5)*	27.3 (2.8)*
CVLT score	6.64 (2.4)	6.10 (2.4)*	5.87 (2.4)*	5.59 (2.3)*	5.32 (2.4)*
STW score	49.94 (5.7)	49.29 (7.1)	50.1 (5.5)	49.54 (8.1)	48.98 (7.6)
SDMT score	50.9 (11.2)	47.30 (11.27)*	46.9 (12.6)*	42.34 (12.57)*	41.44 (12.57)*

SI conversion factors: HbA1c to mmol/mol, multiply values by 0.01; fasting blood glucose to mmol/l, multiply by 0.055

*BMI* body mass index, *CVLT* California Verbal Learning Test, *HbA1c* glycated haemoglobin, *IFG* impaired fasting glucose, *IGT* impaired glucose tolerance, *KDM* known diabetes mellitus, *MMSE* Mini-Mental Status Examination, *NDM* new diabetes mellitus, *NGT* normal glucose tolerance, *SD* standard deviation, *SDMT* Symbol–Digit Modalities test, *STW* Spot the Word

*Significantly different from NGT at *p* <0.05 (with Bonferroni correction)

### Glucose tolerance status as a predictor of cognitive function 7 and 12 years later

Table 2 presents the means of 12-year cognitive tests according to glucose tolerance status at baseline and 5 years. KDM at baseline and at 5-year follow-up was a predictor of slower processing speed at 12 years of follow-up for both age groups. Sex by glucose tolerance status interactions were non-significant for memory and processing speed (data not shown).

**Table 2:** Marginal means for cognitive scores at 12 years of follow-up stratified by age group and diabetes status as baseline and 5-year and 12-year follow-up

	Speed						Memory						Verbal					
	Baseline		5 years		12 years		Baseline		5 years		12 years		Baseline		5 years		12 years	
Glucose tolerance status	Mean (SE)	*N*	Mean (SE)	*N*	Mean (SE)	*N*	Mean (SE)	*N*	Mean (SE)	*N*	Mean (SE)	*N*	Mean (SE)	*N*	Mean (SE)	*N*	Mean (SE)	*N*
25–59 years																		
NGT	52.39 (0.20)	2982	52.09 (0.23)	2506	51.99 (0.25)	2699	6.75 (0.05)	2962	5.23 (0.17)	2492	6.73 (0.61)	2686	50.06 (0.12)	2943	50.09 (0.14)	2480	50.09 (0.15)	2675
IFG	51.71 (0.64)	192	51.86 (0.81)	121	52.06 (0.67)	184	6.84 (0.16)	194	5.50 (0.36)	121	7.04 (0.17)	182	49.57 (0.39)	189	49.67 (0.50)	120	50.0 (0.41)	179
IGT	53.29 (0.54)	287	52.19 (0.68)	177	52.64 (0.53)	309	6.52 (0.13)	287	4.78 (0.27)	178	6.76 (0.13)	307	50.12 (0.33)	283	50.44 (0.42)	174	49.89 (0.33)	300
New diabetes mellitus	50.51 (1.04)	73	51.52 (1.12)	61	51.62 (0.70)	173	6.56 (0.26)	71	5.00 (0.43)*	61	6.34 (0.17)	171	50.44 (0.65)	68	50.64 (0.70)	58	49.59 (0.43)	171
Known diabetes mellitus	49.17 (1.09)	66	36.82 (0.92)	95	49.49 (0.88)	105	6.29 (0.28)	65	4.97 (0.33)	95	6.38 (0.22)	104	49.40 (0.67)	64	48.81 (0.57)	92	49.55 (0.55)	99
60–85 years																		
NGT	38.81 (0.67)	584	37.24 (0.78)	458	39.36 (0.85)	433	5.27 (0.14)	512	6.78 (0.06)	467	5.06 (0.18)	441	50.09 (0.14)	491	51.22 (0.57)	447	51.78 (0.64)	416
IFG	38.60 (1.47)	52	38.98 (1.64)	45	36.44 (1.90)	37	5.40 (0.31)	54	6.80 (0.20)	45	4.46 (0.37)	40	49.07 (0.50)	46	49.12 (1.23)	42	48.92 (1.31)	39
IGT	37.26 (1.03)	127	37.24 (1.22)	97	36.36 (1.18)	111	4.95 (0.22)	127	6.47 (0.17)	48	4.97 (0.25)	113	50.44 (0.42)	120	51.35 (0.92)	85	51.23 (0.90)	107
New diabetes mellitus	35.15 (1.63)	42	38.98 (2.01)	27	37.18 (1.38)	71	5.09 (0.35)	43	6.20 (0.28)	27	5.12 (0.30)	70	50.64 (0.70)	40	52.09 (1.45)	27	51.67 (1.06)	66
Known diabetes mellitus	35.10 (1.61)	41	36.82 (1.51)	27	34.76 (1.44)	63	4.56 (0.36)	39	6.33 (0.23)	51	4.51 (0.31)	63	49.59 (0.57)	40	48.59 (1.11)	52	49.70 (1.09)	61

Models adjusted for age, sex, education, BMI, smoking, exercise time and mean arterial blood pressure. Scores for speed range from 0 to 60; scores for memory range from 0 to 16; and verbal ability ranges from 0 to 60

*BMI* body mass index, *NGT* normal glucose tolerance, *IFG* impaired fasting glucose, *IGT* impaired glucose tolerance, *SE* standard error

**p <0.001, *p <0.05 relative to NGT

### HbA1c and blood glucose as predictors of cognitive function

Models estimating the associations of HbA1c and FPG (measured at baseline, 5 years and 12 years) with cognitive performance at 12 years for participants without diabetes showed significant sex by HbA1c interactions (β = −2.57, standard error (SE) = 1.3, *p* <0.05) and sex by FPG interactions (β = −2.12, SE = 0.73, *p* <0.01), and therefore models were stratified by gender (see Table 3). For males in the younger age group, higher HbA1c at 5 years (β = −0.91, SE = 0.26, *p* <0.01) predicted poorer memory performance. In this group, there were also trends suggesting poorer memory, verbal ability and processing speed in young males with high blood glucose levels (*p* <0.05). Specifically, these were poor memory and verbal ability in those with high baseline HbA1c (memory: β = −0.56, SE = 0.26, *p* <0.05; verbal: β = −1.60, SE = 0.67, *p* <0.05), and high HbA1c at 5 years (verbal: β = −1.58, SE = 0.66, *p* <0.05), reduced processing speed in those with high 12-year HbA1c (β = −1.61, SE = 0.68, *p* <0.05), and high FPG at baseline (β = −1.23, SE = 0.54, *p* <0.05) and 5 years (β = −1.33, SE = 0.53, *p* <0.05); however, these did not reach the conservative criterion for significance (*p* <0.01). There were no associations between HbA1c or FPG and cognition in females or in older males. All models were adjusted for age, education, BMI, smoking, MAP and exercise.

**Table 3:** Associations of baseline and 5-year FPG and HbA1c with 12-year cognitive function in non-diabetic participants stratified by sex

	Speed						Memory						Verbal					
	Males			Females			Males			Females			Males			Females		
	β	(SE)	*n*	β	(SE)	*n*	β	(SE)	*n*	β	(SE)	*n*	β	(SE)	*n*	β	(SE)	*n*
HbA1c, 25–59 years																		
Baseline	–1.52	(1.06)	1192	0.63	(0.95)	1541	–0.56*	(0.26)	1188	–0.20	(0.25)	1534	–1.60*	(0.67)	1196	–0.93	(0.55)	1544
5 years	–1.55	(1.07)	1165	1.30	(0.92)	1501	–0.91**	(0.26)	1161	–0.13	(0.24)	1493	–1.58*	(0.66)	1169	–0.50	(0.53)	1504
12 years	–1.61*	(0.68)	1181	0.19	(0.71)	1526	–0.24	(0.17)	1177	0.35	(0.19)	1518	–0.83	(0.43)	1185	–0.01	(0.41)	1529
HbA1c, 60–85 years																		
Baseline	–0.05	(2.11)	260	–4.82	(2.47)	301	–0.29	(0.49)	262	–0.36	(0.54)	299	0.00	(1.64)	265	–3.32	(1.91)	304
5 years	2.15	(2.10)	251	–1.85	(2.53)	292	0.19	(0.49)	253	–0.42	(0.55)	290	1.17	(1.64)	256	–0.63	(1.88)	295
12 years	0.59	(1.62)	259	–1.62	(1.83)	291	0.24	(0.37)	260	–0.48	(0.41)	291	–0.19	(1.25)	263	–0.31	(1.42)	294
FPG, 25–59 years																		
Baseline	–1.23*	(0.54)	1202	0.97	(0.54)	1556	–0.08	(0.13)	1198	0.02	(0.14)	1549	–0.43	(0.35)	1206	0.35	(0.32)	1559
5 years	–1.33*	(0.53)	1165	0.59	(0.47)	1501	0.02	(0.13)	1161	–0.09	(0.13)	1493	–0.18	(0.33)	1169	0.10	(0.27)	1504
12 years	–0.73	(0.38)	1182	0.27	(0.36)	1528	0.07	(0.09)	1178	0.10	(0.10)	1520	–0.14	(0.24)	1186	0.09	(0.21)	1531
FPG, 60–85 years																		
Baseline	1.40	(1.12)	262	–1.75	(1.27)	301	0.23	(0.26)	264	–0.30	(0.27)	299	–0.58	(0.87)	267	0.14	(0.98)	304
5 years	0.13	(0.89)	251	–0.89	(1.16)	292	–0.15	(0.20)	253	–0.31	(0.25)	290	0.31	(0.69)	256	–0.31	(0.86)	256
12 years	1.28	(0.89)	259	–1.62	(0.87)	291	0.24	(0.20)	260	–0.08	(0.19)	291	–0.19	(0.69)	263	–0.53	(0.68)	294

Models adjusted for age, education, BMI, smoking, exercise time and mean arterial blood pressure

*BMI* body mass index, *FPG* fasting plasma glucose, *HbA1c* glycated haemoglobin, *SE* standard error

*p <0.05; **p <0.01

### Patterns of change in blood glucose over 12 years, and cognitive outcomes

**Fig. 1:**
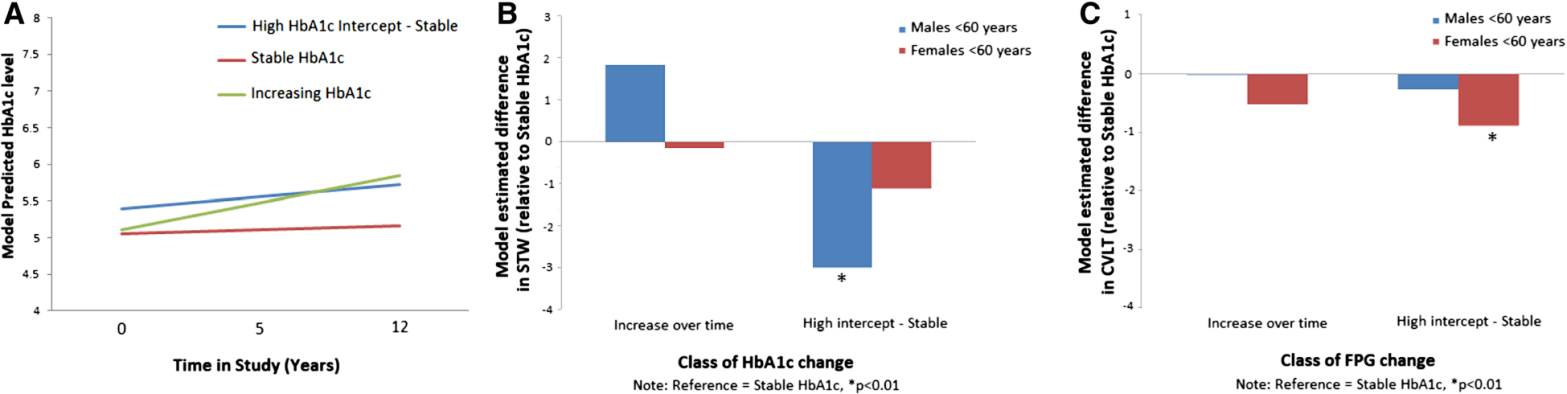
**a**) Latent class analysis identified three main patterns of change in FBG and HbA1c over time. **b**) High-stable HbA1c was associated with decline in verbal skills relative to participants with low stable HbA1c. **c**) High stable FBG was associated with reduced memory performance at 12 years. *CVLT* California Verbal Learning Test, *FPG* Fasting Plasma Glucose, *HbA1c* glycated haemoglobin, *STW* Spot the Word

For individuals without type 2 diabetes, latent class analysis was conducted to identify categories of change in the HbA1c and FBG measures over time. For both age groups, and both blood glucose measures, a three-class outcome fit the data best. Identified classes were described as: (1) normal and stable blood glucose over time (reference); (2) high intercept but stable blood glucose over time; and (3) increasing blood glucose over time. Sample sizes for the ‘high stable’ and ‘increasing’ classes were small relative to the reference group. The three-class outcome for HbA1c for the younger age group is shown in Fig. 1a. For males aged <60 years, models estimating the association between class of HbA1c change and cognition at 12 years revealed that a high, stable pattern of HbA1c levels (*N* = 24) predicted lower verbal ability (β = −2.94, p = 0.008) relative to individuals with normal and stable HbA1c levels (*N* = 1448) (Fig. 1b). This association remained after adjusting for baseline age, education, smoking, BMI, exercise and MAP (β = −3.00, *p* = 0.006). The pattern of change in HbA1c over time did not predict speed of processing or episodic memory at 12 years for young males, and no associations were found for FPG change and cognition (Fig. 1c). For females aged <60 years, high stable FPG (*N* = 27) predicted lower episodic memory at 12 years (β = −1.25, *p* <0.001) relative to the reference group (*N* = 1495) (Fig. 1c), and this remained after adjustment for covariates (β = −0.885, *p* = 0.010). No other associations occurred between pattern of blood glucose over time and cognition. There were no associations between cognitive outcomes and pattern of HbA1c or FPG levels for older males or older females.

### Glucose tolerance status, HbA1c and blood glucose as predictors of global cognitive impairment

The MMSE was administered to individuals aged 60 years and older as a measure of global cognition. In models adjusting for demographics, new diabetes and IFG at baseline were associated with increased risk of global cognitive impairment after 12 years of follow-up. However, these effects become non-significant after adjusting for BMI, level of physical activity and MAP (Additional file 1). HbA1c and FPG were not associated with cognitive impairment (results not shown).

Models examining the association between latent class of HbA1c change and MMSE at 12 years showed a significant relationship between increasing HbA1c levels (*N* = 19) and lower MMSE scores (β = −1.44, *p* <0.01) relative to normal, stable HbA1c levels (*N* = 1543). There was no association between class of FPG change over time and MMSE scores. Models were adjusted for baseline age, education, smoking, BMI, exercise and MAP.

### Sensitivity analyses

Results possibly occurred due to reverse causation, whereby individuals with lower cognitive function are predisposed to develop diabetes. To further evaluate this issue we re-estimated the effect of diabetes status on processing speed, adjusting for 12-year verbal ability since verbal ability changes little over time: baseline (β = −2.635, SE = 1.06, *p* <0.001) and 5 years (β = −1.810, SE = 0.90, *p* = 0.044). KDM remained a risk factor for slower processing speed in the 25–59 year group but the effect in the older group became non-significant. For individuals without diabetes at 12 years, HbA1c at 5 years remained a significant predictor of memory at 12 years for young males (*p* <0.01), and remained non-significant for females and older males. Removal of participants with MMSE <24 did not alter results.

### Adjustment for cardiovascular risk, depression, insulin sensitivity and C-reactive protein

**Fig. 2:**
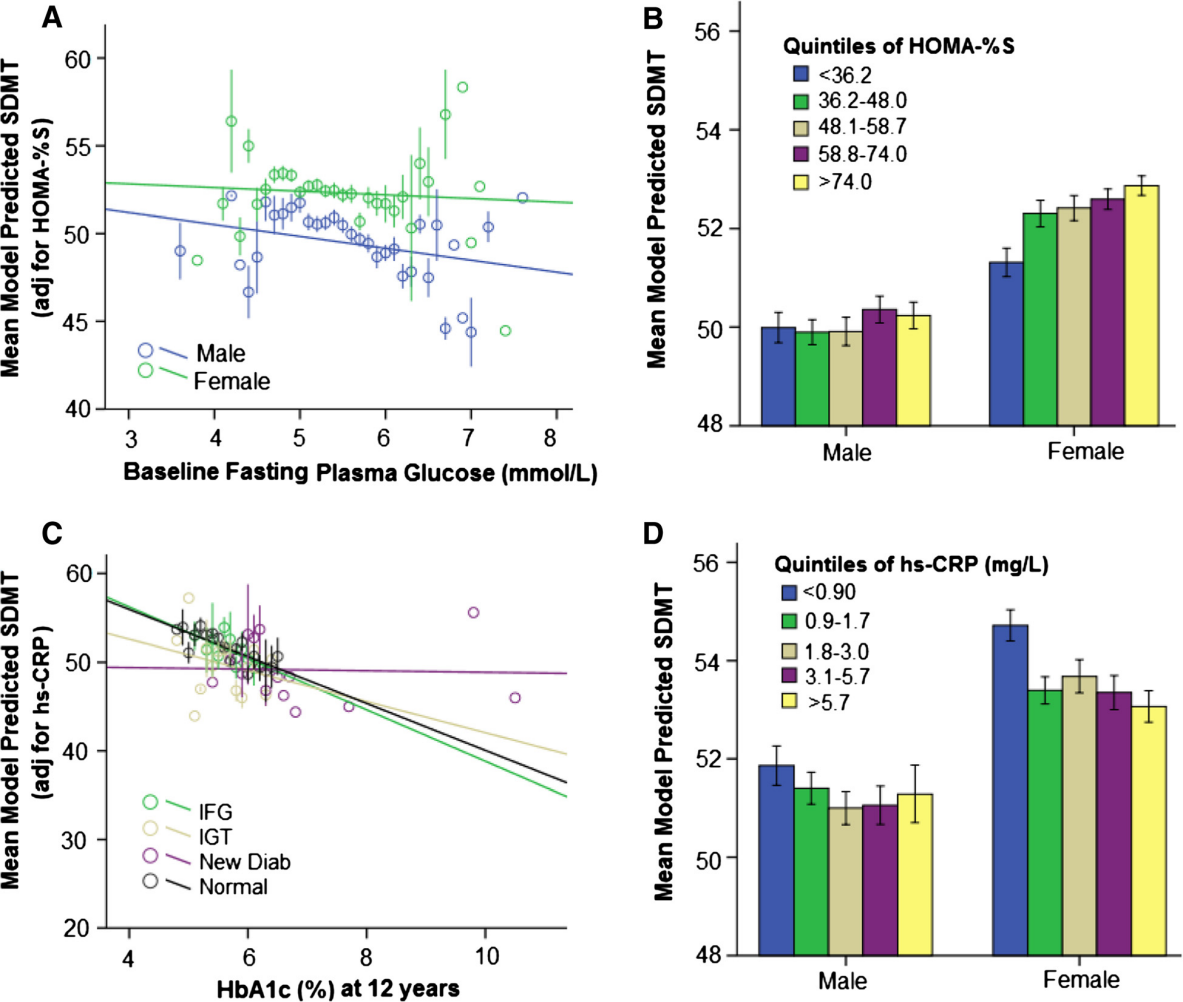
Adjustment for insulin sensitivity and inflammation in models of glycaemia and processing speed (SDMT) at 12 years for participants aged 25–59 years with no known diabetes. **a** Association after adjustment for insulin sensitivity (HOMA-%S) for males and females (25–59 years). **b** Model-predicted SDMT as a function of sex and quintiles of insulin sensitivity (low sensitivity indicates greater insulin resistance). **c** Association after adjustment for hs-CRP (inflammatory marker) as a function of diabetic status. **d** Model-predicted SDMT as a function of sex and quintiles of hs-CRP (higher values indicate greater inflammation). Error bars represent ±1 standard error of the mean. *HbA1c* glycated haemoglobin, *HOMA-2* Homeostatic Model Assessment, *hs-CRP* high-sensitivity C-reactive protein, *IFG* impaired fasting glucose, *IGT* impaired glucose tolerance, *New Diab* new diabetes *SDMT* Symbol–Digit Modalities Test

Significant relationships in the above analyses were further examined by separately adjusting for Framingham cardiovascular disease 10-year percentage risk, depressive symptoms (Center for Epidemiologic Studies Depression Scale- Revised, CESD-R), HOMA-2 insulin sensitivity and hs-CRP (see Additional file 2). All observed associations for young males remained after adjusting for depression and cardiovascular risk. The association between baseline FPG and processing speed in young males was non-significant after adjusting for insulin sensitivity (β = −1.01, SE = 0.59, *p* >0.05). Figure 2a shows this relationship in the HOMA-2 adjusted model for young females and males. Insulin sensitivity was also significantly lower in young males (mean (SE) = 58.9 (0.95)) than females (mean (SE) = 63.4 (0.83), *p* <0.01) (Additional file 2). Figure 2b shows that although there was a trend for lower processing speed with lower insulin sensitivity, predicted SDMT scores were lower across all quintiles of insulin sensitivity in males relative to females. After adjusting for hs-CRP, FPG at 5 years no longer predicted processing speed at 12 years in young males (β = −1.23, SE = 0.67, *p* >0.05). The cross-sectional relationship between HbA1c at 12 years and processing speed was also non-significant in young males (β = −0.74, SE = 0.83, *p* >0.05). Figure 2c shows that adjusting for hs-CRP diminished the relationship most for males with very high HbA1c levels (new diabetes group). Mean CRP levels were significantly higher among young females (mean (SE) = 4.18 (7.85) mg/l) than males (mean (SE) = 3.22 (6.61) mg/l, *p* <0.01). Figure 2d shows that there is nevertheless a trend for reduced processing speed with higher CRP in both males and females. Although elevated CRP was associated with higher blood glucose (new diabetes) in the 25–59 year age group, this was not the case in the older group (data not shown).

## Discussion

This large population-based study examined how change in HbA1C is associated with future cognitive performance, and is one of the few studies to examine the relationship between type 2 diabetes, glycaemia and cognition in both younger and older adults. We found that type 2 diabetes was associated with slower processing speed, after 7 and 12 years of follow-up. This was observed after adjusting for verbal ability, education and other potential confounders and for adults aged younger than 60 years as well as older than 60 years. Among younger men without type 2 diabetes followed for 7 years, we found that higher HbA1C was associated with poorer memory. Trends in the data also suggested that baseline HbA1c and baseline and 5-year FPG were associated with slower processing speed in younger males. Insulin sensitivity and the inflammatory marker hs-CRP contributed to this association in younger males, whereas cardiovascular risk and depressive symptoms did not. No effect of glycaemia on cognition was found for younger females or for older males and females. Young males with persistently high levels of HbA1c over the 12-year study period had lower verbal ability than those with lower, stable HbA1c levels. Young females with persistently high levels of HbA1c had poorer memory at 12 years. Thus, our data suggest that persistently high levels of blood glucose over time in younger adults (<60 years) without type 2 diabetes are associated with lower cognitive performance.

Our findings are consistent with recent results for non-demented older adults showing that diabetes is related to slowed speed of processing [26]. Our data are unable to shed light on causal associations. Possible explanations of our findings include that reduced cognition may impact on self-management of diabetes risk and, by this means, increase the incidence of diabetes. Alternatively, diabetes may lead to structural brain changes which result in cognitive changes [26]. Recent neuroimaging research supports the view that high glucose levels in people without diabetes affects multiple brain sub-regions, including lower grey/white matter regional volumes in the frontal cortices [27]. Our findings are also consistent with findings in older adults linking high non-diabetic blood glucose levels with poor performance in verbal memory and learning [12, 28], reduced hippocampal volume [11, 28] and hippocampal microstructure [12].

Recent randomised controlled trials, however, indicate that strict glycaemic control does not slow the rate of cognitive decline in type 2 diabetes patients when compared with standard diabetes management [29], although brain volume loss was reduced in those undergoing intensive glycaemic control. Management of other factors such as vascular risk factors in target at-risk groups has been proposed as a better approach to preventing cognitive impairment and decline [5].

Sex differences in the association between glycaemia and cognitive function have rarely been investigated [9, 27]. Our finding of more cardiovascular risk factors, higher blood glucose and reduced memory in young, non-diabetic males (see Additional file 1) is consistent with some reports [27] and with broader findings that, in mid-life, males have an elevated risk of vascular disease [30], more cerebral white matter lesions [31], greater insulin resistance and glycaemia [32], and are diagnosed with type 2 diabetes at lower BMI than females [33]. A recent report showed that the medial temporal lobe is particularly sensitive to vascular risk factors in older men compared with women [34]. Although adjustment for cardiovascular risk did not alter the findings, insulin sensitivity—which was also reduced in men relative to women—did influence the link between glycaemia and cognition.

Although only a small number of individuals showed a consistent pattern of change in blood glucose in our sample (i.e., high stable, or increasing), our findings represent the only data to our knowledge on the association between cognition and the pattern of change in blood glucose over time in adults without type 2 diabetes. We found that persistently high levels of FPG and HbA1c over time are a marker of poorer episodic memory in females (decrease of 0.89 words recalled) and poorer verbal ability (decrease of 3.0 words) in males aged <60 years. In contrast to previous work on older adults showing correlated change in MMSE scores with HbA1c levels over time [13], we found no association between the pattern of HbA1c change and cognitive outcome at 12 years in our sample of older adults. The lack of findings for older adults in our study should be interpreted with caution because it may reflect the greater impact of attrition due to death in those with high HbA1c and FBG levels, and greater effect of this biased attrition in the older group than the younger group (see Additional file 3).

In our study, cardiovascular risk did not attenuate the observed link between glycaemia and cognition in men aged younger than 60 (see Additional file 4). Our models also included other vascular risk factors including hypertension (MAP), smoking and BMI—which have been consistently associated with cognitive decrements in the general population and in those in prediabetic stages [5]. In our study, after adjustment for vascular risk factors, only insulin sensitivity and CRP contributed to the observed relationship. There is a growing understanding that insulin resistance may mediate the relationship between chronic high-fat diets and reduced cognition, both in rat models and in humans with type 2 diabetes, while at the same time contributing to cardiovascular disease, depression and hypertension [35]. Recent models of cognitive impairment in type 2 diabetes and prediabetes propose insulin resistance as part of a mechanism leading to microvascular and neuronal metabolic insults within the brain [5, 36]. Apparent from Fig. 2b, however, is that, even among those with high insulin sensitivity, males had slower processing speed than females, suggesting there are other factors contributing to the observed sex difference.

Although adjustment for insulin sensitivity ameliorated the association of HbA1c to processing speed, the relationship between HbA1c and memory remained significant after adjusting for insulin sensitivity. This contrasts with a recent report that insulin resistance predicts temporal lobe atrophy as well as memory performance (Rey's Auditory Verbal Learning Test) in a largely normoglycaemic middle-aged cohort (57.66 ± 6.48 years) [10]. Levels of the inflammatory marker hs-CRP influenced the association between Wave 3 HbA1c and processing speed. Notably, this adjustment appeared to have the greatest effect for men with very high HbA1c (in the new diabetes group), who also had higher hs-CRP levels.

Our study was limited by the lack of longitudinal cognitive data and a measure of executive function. The sample was biased towards healthy and more educated participants, and attrition due to mortality was correlated with elevated glycaemia and diabetes status. Study strengths include the large sample size, inclusion of younger adults and measurement of memory and processing speed, longitudinal data on glucose tolerance status and glucose levels, and relevant covariates.

## Conclusions

Both type 2 diabetes and higher levels of HbA1C among men without diabetes may increase the risk of poor cognitive function and potentially increase the risk of cognitive impairment. Adults with persistently high HbA1C within the normal range are at risk of poorer cognitive function and may be a new target for intervention for prevention of future cognitive decline. Longitudinal research is urgently needed to examine how varying levels and trajectories of HbA1C influence cognitive decline in adulthood so that clinical advice can be developed for optimal cognitive health in ageing.

## Additional files

Additional file 1: Table S1. Presenting baseline cardiovascular, inflammatory, kidney, insulin and blood glucose measures, and depressive symptoms (at 12 years) as a function of sex and age for individuals without diabetes at 12-year follow-up. Baseline characteristics of the sample. Additional file 2: Table S2. Presenting baseline glucose tolerance status and risk of global cognitive impairment. Glucose tolerance status and risk of cognitive impairment as measured by MMSE. Additional file 3: Table S3. Presenting glucose tolerance, HbA1c and fasting blood glucose at baseline and 2005 as a function of death status at 2012. Relationship between attrition due to mortality by 2012 and blood glucose variables at baseline and 2005. Additional file 4: Table S4. Presenting adjustment for covariates in models of blood glucose and cognition—beta values and standard errors. Effect of adjustment for depression, cardiovascular disease risk, insulin sensitivity and C-reactive protein on the association between blood glucose and cognition.

## Abbreviations

AD: Alzheimer’s disease
AusDiab: Australian Diabetes, Obesity and Lifestyle Study
BMI: Body mass index
CVLT: California Verbal Learning Test
FPG: Fasting plasma glucose
GLM: Generalised linear models
HbA1c: Glycated haemoglobin
HOMA-2: Homeostatic model assessment
hs-CRP: High-sensitivity C-reactive protein
IFG: Impaired fasting glucose
IGT: Impaired glucose tolerance
KDM: Known diabetes
MAP: Mean arterial blood pressure
MCI: Mild cognitive impairment
MMSE: Mini-Mental State Examination
NDM: Newly diagnosed diabetes
NGT: Normal glucose tolerance
OGTT: Oral glucose tolerance test
PG: plasma glucose
SDMT: Symbol–Digit Modalities Test
SE: Standard error
STW: Spot the Word
