# Supplementary material for: Association of cognitive function with glucose tolerance and trajectories of glucose tolerance over 12 years in the AusDiab study
Source: Alzheimers Res Ther. 2015 Jul 12;7(1):48. doi: 10.1186/s13195-015-0131-4 (PMC4499451; doi:10.1186/s13195-015-0131-4)
Supplement: Additional file 1: Table S1. — Presenting baseline cardiovascular, inflammatory, kidney, insulin and blood glucose measures, and depressive symptoms (at 12 years) as a function of sex and age for individuals without diabetes at 12-year follow-up. Baseline characteristics of the sample. [file 13195_2015_131_MOESM1_ESM.docx]

Table S1 Baseline cardiovascular, inflammatory, kidney, insulin, and blood glucose measures, and depressive symptoms (at 12 years) as a function of sex and age for individuals without diabetes at 12 year follow-up

|  | 25-59 Year olds | |  | 60 – 85 year olds | |  |
| --- | --- | --- | --- | --- | --- | --- |
|  | Male | Female |  | Male | Female |  |
| Smoking status (N(%)) Current | 199 (13.5%) | 204 (10.4%) | ** | 19 (6.0%) | 13 (3.7%) | NS |
| Ex-smoker | 440 (29.9%) | 493 (25.2%) | ** | 146 (46.3%) | 67 (19.3%) | ** |
| BMI value (kg/m.2) | 26.74 (3.77) | 25.76 (5.01) | ** | 26.49 (2.97) | 26.45 (4.57) | NS |
| exercise time (minutes) † | 338.95 (371.23) | 245.89 (294.35) | ** | 337.04 (357.31) | 275.79 (308.75) | * |
| Mean arterial pressure | 91.53 (10.54) | 83.90 (10.74) | ** | 96.67 (11.35) | 89.88 (12.90) | ** |
| Cholesterol (mmol/L) | 5.66 (1.00) | 5.43 (1.02) | ** | 5.66 (0.99) | 6.06 (0.95) | ** |
| High Density Lipoprotein (mmol/L) | 1.27 (0.29) | 1.59 (0.36) | ** | 1.33 (0.34) | 1.64 (0.39) | ** |
| Low Density Lipoprotein (mmol/L) | 3.66 (0.88) | 3.30 (0.90) | ** | 3.64 (0.89) | 3.75 (0.89) | NS |
| Triglycerides (mmol/L) | 1.61 (1.08) | 1.18 (0.73) | ** | 1.55 (0.91) | 1.49 (0.83) | NS |
| Inflammatory markers |  |  |  |  |  |  |
| Fibrinogen (mmol/L) † | 3.34 (0.74) | 3.58 (0.82) | ** | 3.62 (0.76) | 3.67 (0.70) | NS |
| Wave 2 hs C-reactive protein (mg/L) † | 3.22 (6.61) | 4.18 (7.85) | ** | 3.82 (7.07) | 4.49 (8.69) | NS |
| Glucose metabolism |  |  |  |  |  |  |
| Fasting Plasma Blood Glucose (mmol/L) | 5.47 (0.45) | 5.15 (0.44) | ** | 5.53 (0.47) | 5.35 (0.46) | ** |
| HbA1c calculated from Total GHb | 5.08 (0.24) | 5.00 (0.25) | ** | 5.18 (0.26) | 5.17 (0.24) | NS |
| Insulin Sensitivity (HOMA-%S) | 58.9(0.95) | 63.4(0.83) | ** | 60.85(2.25) | 58.0(1.29) | NS |
| Chronic Kidney Disease (eGFR<60) † | 11(0.7%) | 84(4.2%) | ** | 32(10.0%) | 84(24.0%) | ** |
| Wave 3 Depressive symptoms (CESD) † | 3.66 (4.17) | 4.22 (4.57) | ** | 3.57 (3.79) | 4.36 (4.30) | * |

*p<0.05, **p<0.01; † Females worse than males
