# Supplementary material for: Association of cognitive function with glucose tolerance and trajectories of glucose tolerance over 12 years in the AusDiab study
Source: Alzheimers Res Ther. 2015 Jul 12;7(1):48. doi: 10.1186/s13195-015-0131-4 (PMC4499451; doi:10.1186/s13195-015-0131-4)
Supplement: Additional file 2: Table S2. — Presenting baseline glucose tolerance status and risk of global cognitive impairment. Glucose tolerance status and risk of cognitive impairment as measured by MMSE. [file 13195_2015_131_MOESM2_ESM.docx]

Table S2 Baseline glucose tolerance status and risk of global cognitive impairment (measured by MMSE) at 12 years (n = 1798)

|  | Model 1 | Model 2 |
| --- | --- | --- |
|  | OR (CI 95%) | OR (CI 95%) |
| Normal Glucose Tolerance | reference | reference |
| Impaired Fasting Glucose | 2.02 (1.02, 4.58) | 1.77(0.82, 3.85) |
| Impaired Glucose Tolerance | 1.70 (0.89, 3.24) | 1.11(0.55, 2.58) |
| New diabetes | 2.59 (1.08, 6.24) | 1.53 (0.58. 4.03) |
| Known Diabetes Mellitus | 1.40 (0.50, 4.49) | 1.10 (0.36, 3.41) |

Note. Reference group is normal glucose tolerance. Model 1 adjusts for age, sex and education. Model 2 adjusts for age, sex, education, BMI, physical activity time, MAP. MMSE – Mini-Mental Status Exam; OR – Odds Ratio.
