# Supplementary material for: Association of cognitive function with glucose tolerance and trajectories of glucose tolerance over 12 years in the AusDiab study
Source: Alzheimers Res Ther. 2015 Jul 12;7(1):48. doi: 10.1186/s13195-015-0131-4 (PMC4499451; doi:10.1186/s13195-015-0131-4)
Supplement: Additional file 3: Table S3. — Presenting glucose tolerance, HbA1c and fasting blood glucose at baseline and 2005 as a function of death status at 2012. Relationship between attrition due to mortality by 2012 and blood glucose variables at baseline and 2005. [file 13195_2015_131_MOESM3_ESM.docx]

Table S3 Adjustment for covariates in models of blood glucose and cognition -Beta values and standard errors

|  | 25-59 years | | | | 60-85 years | | | |
| --- | --- | --- | --- | --- | --- | --- | --- | --- |
|  | Male | | Female | | Male | | Female | |
|  | β | (SE) | β | (SE) | β | (SE) | β | (SE) |
| Model 1 |  |  |  |  |  |  |  |  |
| CVLT – W1 HbA1c | -0.56* | (0.26) | -0.20 | (0.25) | -0.29 | (0.49) | -0.36 | (0.54) |
| CVLT – W2 HbA1c | -0.91** | (0.26) | -0.13 | (0.24) | 0.19 | (0.49) | -0.42 | (0.55) |
| SDMT – W3 HbA1c | -1.61* | (0.68) | 0.19 | (0.71) | 0.59 | (1.62) | -1.62 | (1.83) |
| SDMT – W1 FPG | -1.23* | (0.54) | 0.97 | (0.54) | 1.40 | (1.12) | -1.75 | (1.27) |
| SDMT – W2 FPG | -1.33* | (0.53) | 0.59 | (0.47) | 0.13 | (0.89) | -0.89 | (1.16) |
| Model 2 (Depression) | | | | | | | | |
| CVLT – W1 HbA1c | -0.56* | 0.26 | -0.21 | 0.25 | -0.31 | 0.49 | -0.37 | 0.55 |
| CVLT – W2 HbA1c | -0.92** | 0.26 | -0.12 | 0.25 | 0.15 | 0.50 | -0.36 | 0.56 |
| SDMT – W3 HbA1c | -1.56* | 0.69 | 0.10 | 0.71 | 0.35 | 1.61 | -2.11 | 1.83 |
| SDMT – W1 FPG | -1.20* | 0.54 | 0.95 | 0.54 | 1.08 | 1.11 | -1.95 | 1.28 |
| SDMT – W2 FBG | -1.27* | 0.54 | 0.55 | 0.47 | -0.20 | 0.88 | -0.20 | 0.88 |
| Model 3 (CVD risk) |  |  |  |  |  |  |  |  |
| CVLT – W1 HbA1c | -0.53* | 0.26 | -0.17 | 0.26 | -0.12 | 0.49 | -0.24 | 0.55 |
| CVLT – W2 HbA1c | -0.89** | 0.26 | -0.07 | 0.25 | 0.40 | 0.49 | -0.24 | 0.55 |
| SDMT – W3 HbA1c | -1.60* | 0.68 | 0.29 | 0.72 | 1.34 | 1.61 | -1.35 | 1.89 |
| SDMT – W1 FPG | -1.18* | 0.55 | 1.09 | 0.55 | 1.45 | 1.10 | -1.63 | 1.32 |
| SDMT – W2 FPG | -1.32* | 0.5 | 0.68 | 0.48 | 0.58 | 0.89 | -0.58 | 1.22 |
| Model 4 (HOMA-%S) | | | | | | | | |
| CVLT – W1 HbA1c | -0.72** | 0.27 | -0.08 | 0.27 | -0.27 | 0.49 | -0.43 | 0.55 |
| CVLT – W2 HbA1c | -0.93** | 0.26 | -0.07 | 0.25 | 0.18 | 0.49 | -0.49 | 0.56 |
| SDMT – W3 HbA1c | -1.61* | 0.68 | 0.18 | 0.71 | 0.69 | 1.61 | -1.64 | 1.85 |
| **SDMT – W1 FPG** | **-1.01** | **0.59** | 0.61 | 0.59 | 1.37 | 1.12 | -2.40 | 1.33 |
| SDMT – W2 FPG | -1.36* | 0.55 | 0.62 | 0.49 | -0.08 | 0.89 | -1.31 | 1.25 |
| Model 5 (CRP) |  |  |  |  |  |  |  |  |
| CVLT – W1 HbA1c | -0.66* | 0.32 | -0.37 | 0.33 | 0.29 | 0.62 | 0.05 | 0.68 |
| CVLT – W2 HbA1c | -1.16** | 0.30 | -0.42 | 0.30 | -0.01 | 0.58 | -0.77 | 0.65 |
| **SDMT – W3HbA1c** | **-0.74** | **0.83** | -0.48 | 0.87 | 0.84 | 1.98 | **-5.30*** | **2.30** |
| SDMT – W1 FPG | -1.77** | 0.67 | 0.50 | 0.66 | 1.09 | 1.41 | -1.86 | 1.55 |
| **SDMT – W2 FPG** | **-1.23** | **0.67** | 0.21 | 0.56 | 0.14 | 1.04 | -1.69 | 1.39 |

Note.

*p <0.05, ** p <0.01

Model 1: Age, education, smoking status, BMI, exercise time, MAP.

Model 2: Model 1 + Wave 3 depression score (CESD score).

Model 3: Model 1 + Cardiovascular disease risk 10 year based on diastolic BP

Model 4: Model 1 + Wave 1 and Wave 2 Insulin sensitivity (HOMA-%S)

Model 5: Model 1 + Wave 2 CRP (high sensitivity c reactive protein)

Bold: association between glucose and cognition for young males non-significant after adjustment.
