# Supplementary material for: Association of cognitive function with glucose tolerance and trajectories of glucose tolerance over 12 years in the AusDiab study
Source: Alzheimers Res Ther. 2015 Jul 12;7(1):48. doi: 10.1186/s13195-015-0131-4 (PMC4499451; doi:10.1186/s13195-015-0131-4)
Supplement: Additional file 4: Table S4. — Presenting adjustment for covariates in models of blood glucose and cognition—beta values and standard errors. Effect of adjustment for depression, cardiovascular disease risk, insulin sensitivity and C-reactive protein on the association between blood glucose and cognition. [file 13195_2015_131_MOESM4_ESM.docx]

Table S4 Glucose tolerance, HbA1c and fasting blood glucose at baseline and 2005 as a function of death status in 2012.

|  | NGT | IFG | IGT | NDM | KDM | HbA1c | FBG |
| --- | --- | --- | --- | --- | --- | --- | --- |
|  | N (%) | N (%) | N (%) | N (%) | N (%) | Mean (SD) | Mean (SD) |
| Baseline |  |  |  |  |  |  |  |
| Alive 2012 | 7466 (67.6%)*** | 568 (5.1%) | 1109 (10.0%)*** | 349 (3.2%) | 289 (2.6%)*** | 5.18 (0.57)*** | 5.52 (1.02)*** |
| Dead 2012 | 641 (1.1%) | 82 (0.7%) | 269 (2.4%) | 119 (1.1%) | 152 (1.4%) | 5.52 (0.94) | 6.03 (1.77) |
| 2005 |  |  |  |  |  |  |  |
| Alive 2012 | 4645 (73.3%) | 284 (4.5%) | 505 (8.0%)*** | 182 (2.9%) | 321(5.1%)*** | 5.46 (0.52)*** | 5.47 (1.00)*** |
| Dead 2012 | 239 (3.8%) | 19 (0.3%) | 76 (1.2%) | 20 (0.3%) | 50 (0.8% | 5.67(0.65) | 5.70 (1.29) |

Note - *** p<001. % represents proportion of total N – not proportion dead at 2012. Those with Diabetes Type 1 not included in analysis.
